# Tuberculosis and tuberculosis-human immunodeficiency virus in post-conflict West Africa: Lessons for integrated surveillance from 2000 to 2022

**DOI:** 10.4102/jphia.v17i1.1700

**Published:** 2026-05-30

**Authors:** John Kwame Duah

**Affiliations:** 1 Department of Political Science, Health Services Administration Program, Auburn University, Auburn, Alabama, United States of America

**Keywords:** tuberculosis, TB-HIV co-infection, fragile settings, integrated surveillance, health security, fixed-effects, Liberia, Sierra Leone

## Abstract

**Background:**

Post-conflict health systems experienced surveillance and delivery challenges that hindered tuberculosis (TB) and TB-human immunodeficiency virus (HIV) control. Recent evidence shows that fragile and conflict-affected health systems still lack integrated TB-HIV services, routine testing, and reliable data, delaying lower incidence rates even as case detection rises.

**Aim:**

This study examined TB and TB-HIV dynamics in Liberia and Sierra Leone to identify practice-oriented lessons for integrated surveillance and system resilience.

**Setting:**

Study was conducted on data from Liberia and Sierra Leone, 2000–2022.

**Methods:**

A two-country annual panel was assembled from World Health Organisation (WHO) TB indicators, World Bank gross domestic product (GDP) and health expenditure per capita, and conflict events. Primary estimation used two-way fixed effects with Driscoll-Kraay inference, and robustness checks used linear mixed-effects and ordinary least squares (OLS) with Heteroskedasticity–consistent standard error estimator, type 3 (HC3). A 2016–2022 subset assessed TB-HIV testing coverage. Effect sizes with Driscoll-Kraay 95% confidence intervals (CIs) were emphasised.

**Results:**

Across specifications, higher GDP per capita was consistently associated with lower TB incidence. Tuberculosis-human immunodeficiency virus co-infection indicators showed varied associations. In 2016–2022, TB-HIV testing coverage was positively associated with TB incidence, suggesting that expanded testing may increase detection before incidence declines. Health expenditure per capita was similarly positively associated with incidence in 2016–2022, consistent with higher spending during periods of increased case detection. Results were robust across alternative estimators, and standard collinearity diagnostics did not indicate problematic multicollinearity.

**Conclusion:**

In post-conflict settings, strengthening economic capacity, integrating TB-HIV services, and improving surveillance quality were foundational for sustained TB control. Short-run increases in detected cases should be anticipated as testing expands. Programme resources, integration, and data systems were needed to convert detection gains into incidence reduction.

**Contribution:**

The study identified practice-oriented lessons for integrated surveillance and system resilience in post-conflict settings.

## Introduction

Tuberculosis (TB) is a major public-health threat in fragile and post-conflict settings, where systemic instability undermines effective disease control. It remains one of the most persistent and lethal infectious diseases worldwide, causing an estimated 10.8 million new cases and 1.25m deaths in 2023, including 161 000 among people with human immunodeficiency virus (HIV) infection.^1^ Despite international initiatives, efforts to control TB have faltered^2^ and progress is inconsistent.^3^ In fragile and post-conflict states, under-resourced health systems,^2^ fragmented surveillance infrastructure,^2^ and limited access to diagnostic and treatment services leave populations acutely vulnerable.^4^ In such settings, TB-HIV co-infection has emerged as an intensifying syndemic that heightens risk for individuals and populations. This co-epidemic complicates case management and further strains already overburdened health systems.^5^ The World Health Organisation (WHO) warns that without urgent, integrated TB-HIV testing and treatment strategies, especially in high-burden settings with weakened system resilience, the human toll will intensify.^6^

Liberia and Sierra Leone, two West African nations recovering from prolonged civil conflict and the 2014–2016 Ebola outbreak, illustrate the intersection of post-conflict recovery and infectious disease vulnerability.^7,8,9^ Both countries experienced significant disruptions to health-service delivery,^8^ including TB programme setbacks during the Ebola crisis, followed by targeted recovery efforts.^10^ Tuberculosis incidence and TB-HIV co-infection continue to affect both countries,^11^ and the trends in their rates have grown more dissimilar over time. For instance, from 2000 to 2022, Liberia’s TB incidence rose from approximately 240 to more than 300 cases per 100 000 population, with a recent plateau, whereas Sierra Leone’s incidence declined from 318 to 283 per 100 000.^11^ These contrasting patterns motivate examination of economic conditions, health-system recovery and programme integration as potential drivers of TB outcomes in fragile settings.

This study analyses how two post-conflict countries with similar backgrounds have addressed the dual burden of TB-HIV while operating under limited resources. Prior research links structural determinants such as gross domestic product (GDP) per capita, health expenditure and conflict exposure to TB outcomes.^12,13,14^ However, few studies compare fragile states using designs that track change over time. Although TB-HIV testing coverage is a key intervention point,^15^ evidence on its relationship with TB incidence and mortality remains limited,^16^ particularly where surveillance systems are being rebuilt.^17^

A retrospective ecological panel design was employed, utilising publicly available WHO data from 2000 to 2022 to characterise patterns in TB incidence, TB-HIV co-infection rates, and TB mortality (excluding deaths attributable to HIV) in Liberia and Sierra Leone. Regression analyses were conducted to assess relationships between TB outcomes and major variables such as GDP per capita, health expenditure per capita, TB-HIV co-infection and the frequency of conflict events. For the years 2016–2022, an additional analysis evaluated the association between TB-HIV testing coverage and TB incidence.

The integration of epidemiological data and contextual factors provides evidence to support the strengthening of integrated disease surveillance and system resilience in fragile settings. The analytical approach draws on three frameworks: health security and disease surveillance in fragile states, including core capacities in the International Health Regulations (IHR); syndemic theory, which conceptualises TB-HIV as a biosocial interaction that heightens vulnerability in post-conflict populations^5^; and health systems strengthening, with emphasis on intervention and system indicators such as TB-HIV testing coverage,^16^ health expenditure^14^ and economic recovery.^18^ The objective of this study was to describe and compare TB incidence trends and TB-HIV co-infection and testing coverage in Liberia and Sierra Leone from 2000 to 2022, and to assess whether TB-HIV testing coverage and selected economic and conflict indicators were associated with TB incidence and TB mortality that excludes HIV-attributable deaths, with the expectation that TB incidence would decline over time and that higher TB-HIV testing coverage would be associated with lower TB incidence in 2016–2022.

## Research methods and design

### Study design

This retrospective ecological panel study included country–year observations for Liberia and Sierra Leone from 2000 to 2022. There were 46 observations in total and 14 for the 2016–2022 subset.

### Setting

Liberia and Sierra Leone served as the study settings. The period was 2000–2022, with a focused subset for 2016–2022 when TB-HIV testing coverage was available.

### Study population and sampling strategy

Data from all recorded country–year combinations for Liberia and Sierra Leone were utilised, with no sampling procedures applied. Analyses for each period were conducted using only complete cases.

### Data collection

TB indicators were obtained from the WHO Global Tuberculosis Database, including incidence rate, mortality not attributable to HIV, case notification rate, case detection rate, TB-HIV co-infection and TB-HIV testing coverage. Macroeconomic variables, specifically GDP per capita in constant 2015 US dollars and current health expenditure per capita in US dollars ($), were sourced from the World Bank World Development Indicators. Conflict event data were aggregated by country and year from Armed Conflict Location and Event Data (ACLED). Operational definitions, coding and measurement approaches, variable types, and data sources are detailed in Table 1. Tuberculosis-human immunodeficiency virus testing coverage data are available for 2016–2022.

**TABLE 1:** Operational definitions, coding/measurement, variable type and data sources.

Variable	Operational definition	Coding/measurement	Variable type	Data source
TB incidence rate	Annual number of new and relapse TB cases per 100 000 population	Numeric rate per 100 000; WHO modelled country–year estimate	Continuous (rate)	WHO Global Tuberculosis Report/ Global TB Database
TB-HIV co-infection (%)	Percentage of notified TB cases that are HIV-positive in a given year	Per cent (%); proportion of TB cases co-infected with HIV	Continuous (per cent)	WHO TB-HIV Surveillance/Global TB Database
TB-HIV testing coverage (%)	Percentage of notified TB cases with a documented HIV test result (known HIV status) in a given year	Per cent (%); (TB cases with known HIV status ÷ notified TB cases) × 100	Continuous (per cent)	WHO Global TB Database (TB-HIV module). Available 2016–2022
TB mortality rate	Annual TB deaths (excluding HIV-attributable) per 100 000 population	Numeric rate per 100 000; WHO modelled mortality estimate	Continuous (rate)	WHO Global Health Estimates/Global TB Database
TB case notification rate	Number of new and relapsed TB cases notified to national programmes per 100 000 population	Numeric rate per 100 000; derived from WHO notification data	Continuous (rate)	WHO TB Notification Database
TB case detection rate (%)	Proportion of estimated incident TB cases that were notified	Per cent (%); notified cases ÷ estimated incident cases × 100	Continuous (per cent)	WHO TB Estimates and Notification Data
Year (centred)	Derived year index used for descriptive summaries; not entered as a regressor (models include year fixed effects)	Calendar year minus 2000	Continuous (numeric)	Derived from study period
Health expenditure per capita	Total current health expenditure per person	US dollars per capita; annual current health spending ÷ mid-year population	Continuous (currency)	World Bank World Development Indicators (WDI): SH.XPD.CHEX.PC.CD)
GDP per capita	Gross domestic product per person	Constant 2015 US dollars per capita	Continuous (currency)	World Bank WDI
HIV prevalence (%)	Prevalence of HIV among adults aged 15–49 years	Per cent of adults aged 15–49 years living with HIV	Continuous (per cent)	World Bank WDI (WDI: SH.DYN.AIDS.ZS)
Population density	Number of people per square kilometre of land area	People per km^2^ of land area	Continuous (rate)	World Bank WDI WDI: EN.POP.DNST
Urban population (%)	Share of population living in urban areas	Per cent of total population residing in urban areas	Continuous (per cent)	World Bank WDI SP.URB.TOTL.IN.ZS
Hospital beds per 1000	Hospital bed capacity	Number of beds per 1000 population	Continuous (rate)	World Bank WDI (WDI: SH.MED.BEDS.ZS)
Conflict events	Total number of political-violence events recorded in a country–year	Integer count of all ACLED events recorded in the year	Discrete (count)	ACLED Project; author’s country–year aggregation
Conflict fatalities	Total number of deaths from political-violence events in a country-year	Integer sum of ACLED fatalities in the year	Discrete (count)	ACLED; author’s country-year aggregation
Battles	Number of ACLED events classified as Battles in a country–year	Integer count where event_type = Battles	Discrete (count)	ACLED; author’s country–year aggregation
Violence against civilians	Number of ACLED events classified as Violence against civilians’ in a country–year	Integer count where event_type = Violence against civilians	Discrete (count)	ACLED; author’s country–year aggregation
Protests	Number of events classified as Protests in a country-year	Integer count of events with type Protests	Discrete (count)	ACLED

Note: All data sources cover Liberia and Sierra Leone for 2000–2022 unless otherwise specified. Armed Conflict Location and Event Data variables are aggregated from event-level records to the country-year level for analysis. Where applicable, indicator codes from WDI are provided in parentheses for clarity.

GDP, gross domestic product; TB, tuberculosis; HIV, human immunodeficiency virus; WHO, World Health Organisation; ACLED, Armed Conflict Location and Event Data.

### Data preparation and management

Datasets were aligned using International Organization for Standardization (ISO3) country codes and calendar years before being combined into a balanced panel for both countries. Tuberculosis-human immunodeficiency virus testing coverage was calculated as the proportion of notified TB cases with a known HIV status, with values restricted to the 0% – 100% range following data quality assessments. Analyses for each period relied on complete cases only, and no data imputation was undertaken.

### Data analysis

The primary analyses employed two-way fixed-effects models incorporating country and year indicators, estimated using the within transformation to account for unobserved country characteristics and year-specific influences common to both countries. Standard errors were adjusted for both serial and cross-sectional dependence using Driscoll-Kraay heteroskedasticity- and autocorrelation-consistent (HC1) estimators with a Bartlett kernel and a plug-in bandwidth. Given the small sample size (panel with only two countries, *G* = 2), statistical inference was limited. Therefore, emphasis was placed on effect sizes (coefficients) and their 95% Driscoll-Kraay confidence intervals (CIs). When *p*-values were presented in the main text, they were calculated using a conservative *t* reference distribution with degrees of freedom set to *df = G* – 1 = 1.

Models incorporating TB-HIV testing coverage were restricted to the 2016–2022 period, while analyses spanning the entire period (2000–2022) did not include this variable. Sensitivity analyses comprised linear mixed-effects regressions with a random intercept for country and fixed effects for year, as well as pooled ordinary least squares (OLS) models with country and year fixed effects and heteroskedasticity-robust (Heteroskedasticity–consistent standard error estimator, type 3 [HC3]) standard errors. As a result of the limited number of countries, cluster-robust CR2 standard errors were also assessed but yielded unstable estimates. As a result, HC3 standard errors are reported for the OLS robustness checks. All statistical analyses were performed using R version 4.4.2, employing plm (for two-way fixed effects and Driscoll-Kraay estimation via vcovSCC), lmtest (for inference), sandwich (for HC1 and HC3), lme4 and lmerTest packages. In Online Appendix 1 Table 1-A1–Table 4-A1, *p*-values are based on the asymptotic normal reference, and Driscoll-Kraay 95% CIs can be calculated as the coefficient estimate plus or minus 1.96 times the standard error reported.

### Ethical considerations

Only publicly available, de-identified aggregate data were used in this research. Consistent with United States (US) federal regulations (45 CFR 46.104(d)(4)), the study did not constitute human or animal subjects research and therefore did not require approval from an institutional review board.

## Results

### Descriptive statistics summary

Liberia and Sierra Leone each provided data for 23 country-year periods, resulting in a total of 46 observations. The average TB incidence was 287.6 per 100 000 people in Liberia and 307.9 per 100 000 in Sierra Leone, with a combined mean of 297.7 per 100 000 (see Table 2). The mean TB-HIV co-infection rate was 22.1% for Liberia and 15.6% for Sierra Leone. Additional summary statistics are presented in Table 2.

**TABLE 2:** Descriptive statistics (mean and standard deviation) of study variables for Liberia (*n* = 23), Sierra Leone (*n* = 23) and overall sample (*N* = 46), 2000–2022.

Variable	Liberia				Sierra Leone				Overall			
	*n*	%	Mean	s.d.	*n*	%	Mean	s.d.	*n*	%	Mean	s.d.
TB incidence rate (per 100 000)	-	-	287.6	23.9	-	-	307.9	9.9	-	-	297.7	20.8
TB-HIV co-infection (%)	22.1	7.9	-	-	15.6	6.2	-	-	18.9	7.8	-	-
TB mortality rate (per 100 000)	-	-	84.1	12.3	-	-	74.1	26.4	-	-	79.1	21.0
TB case notification rate (per 100 000)	-	-	132.8	36.4	-	-	171.5	47.5	-	-	152.6	46.3
TB case detection rate (%)	45.5	10.7	-	-	56.0	16.5	-	-	50.9	14.8	-	-
Year (centred)	-	-	11.0	6.8	-	-	11.0	6.8	-	-	11.0	6.7
Health expenditure per capita (US$)	-	-	45.2	28.7	-	-	48.6	31.5	-	-	46.9	29.8
GDP per capita (2015 US$)	-	-	641.9	70.4	-	-	927.5	124.4	-	-	784.7	175.6
HIV prevalence (%)	1.6	0.4	-	-	1.6	0.1	-	-	1.6	0.3	-	-
Population density (people/km^2^)	-	-	43.0	8.3	-	-	88.8	15.6	-	-	65.9	26.2
Urban population (%)	48.4	2.7	-	-	39.4	2.6	-	-	43.9	5.2	-	-
Hospital beds per 1000	-	-	1.1	0.5	-	-	0.4	–	-	-	1.0	0.5
Conflict events (count)	-	-	66.0	58.1	-	-	57.8	107.1	-	-	61.9	85.3
Conflict fatalities (count)	-	-	26.2	66.6	-	-	5.7	7.7	-	-	15.9	48.0
Violence against civilians (count)	-	-	11.0	10.2	-	-	8.5	15.4	-	-	9.7	12.9
Protests (count)	-	-	17.0	17.7	-	-	6.0	7.9	-	-	11.5	14.6
TB-HIV testing coverage (%)	82.4	10.7	-	-	98.5	1.1	-	-	90.5	11.1	-	-

Note: ‘Battles’ is omitted because it recorded zero events in both countries from 2000 to 2022. Tuberculosis-human immunodeficiency virus testing coverage (%) is available only for 2016–2022 (*n* = 7 per country); all other variables use 2000–2022 (*n* = 23). A dash (–) indicates that the standard deviation could not be computed because of data being available for only one year.

s.d., standard deviation; TB, tuberculosis; HIV, human immunodeficiency virus; GDP, gross domestic product.

### Temporal trends in tuberculosis incidence

**FIGURE 1:**
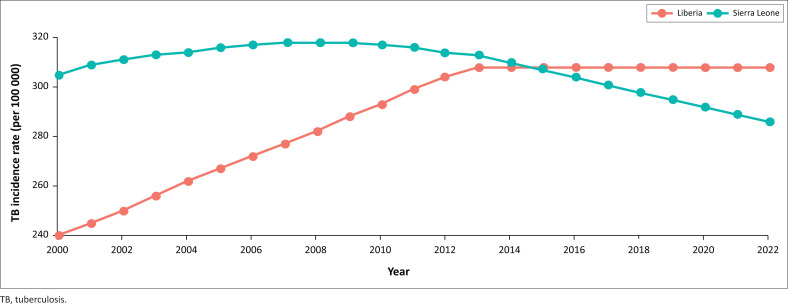
Annual tuberculosis incidence rates in Liberia and Sierra Leone, 2000–2022. Estimates are from the World Health Organisation Global Tuberculosis Database, with 23 yearly observations for each country.

Liberia’s estimated incidence rose from about 240 per 100 000 in 2000 to a peak near 308 in 2013 and then levelled through 2022. Sierra Leone started higher, increased modestly to about 318 in 2008, and declined to 283 by 2022. The post-2008 divergence suggests different epidemic trajectories and implementation timing of control efforts (Figure 1).

#### Primary regression results, 2000–2022

Fixed-effects regression models using Driscoll-Kraay standard errors indicated that GDP per capita was negatively correlated with TB incidence (*b* = –0.145). Tuberculosis-human immunodeficiency virus co-infection also exhibited a negative association with TB incidence (*b* = –1.760). The effect of health expenditure was minimal (*b* = 0.014), and the influence of conflict events was small and negative (*b* = –0.047). The full coefficient estimates and CIs are provided in Table 3.

**TABLE 3:** Two-way fixed-effects regression of tuberculosis incidence rate (per 100 000) in Liberia and Sierra Leone, 2000–2022 (Driscoll-Kraay SEs).

Predictor	*B*	SE (DK)	*T*	*P*
TB-HIV co-infection (%)	-1.760	0.454	-3.880	0.161
Health expenditure per capita (US$)	0.014	0.138	0.105	0.934
GDP per capita (2015 US$)	-0.145	0.018	-7.903	0.080
Conflict events (count)	-0.047	0.035	-1.364	0.403

Note: The dependent variable is TB incidence, measured as cases per 100 000 population. All models adjust for country and year fixed effects. Standard errors are calculated using the Driscoll-Kraay (HC1) approach with a Bartlett kernel and plug-in bandwidth. The analytic sample comprises Liberia and Sierra Leone for the years 2000–2022 (*n* = 46 country-year observations). *P*-values are based on a conservative *t*-distribution with 1 degree of freedom. Interpretation focuses on coefficient estimates and Driscoll-Kraay 95% confidence intervals, which can be derived as the estimate plus or minus 1.96 times the reported standard error.

TB, tuberculosis; HIV, human immunodeficiency virus; GDP, gross domestic product; SE, standard error; DK, Driscoll-Kraay (standard errors).

#### Subset re-estimation, 2016–2022

With variables rescaled, an increase of $100.00 in GDP per capita corresponded to 9.87 fewer TB cases per 100 000 population (95% CI: –11.98 to –7.76). A $100 rise in current health expenditure per capita was linked to 8.87 additional cases per 100 000 (95% CI: 6.68 to 11.06). For TB-HIV testing coverage, a 10-percentage point increase was associated with 0.87 more cases per 100 000 (95% CI: 0.43 to 1.31). Associations for co-infection and conflict events lacked precision. The comprehensive results are presented in Table 4.

**TABLE 4:** Two-way fixed-effects regression estimates for tuberculosis incidence (cases per 100 000), 2016–2022, with Driscoll-Kraay standard errors.

Predictor	*B*	SE (DK)	*t*	95% CI (DK)	*P*
TB-HIV co-infection (per 10 percentage points)	1.390	1.632	0.852	-1.808, 4.589	0.551
Health expenditure per capita (per $100.00)	8.873	1.117	7.942	6.683, 11.062	0.080
GDP per capita, 2015 US$ (per $100.00)	-9.870	1.076	-9.173	-11.979, -7.762	0.069
Conflict events (per 100 events)	-0.150	0.631	-0.237	-1.386, 1.087	0.852
TB-HIV testing coverage (per 10 percentage points)	0.873	0.225	3.883	0.432, 1.314	0.160

Note: Estimates are from two-way fixed-effects models with Driscoll-Kraay (HC1) standard errors. Each coefficient (B) reflects the change in TB incidence per 100 000 population associated with the specified unit increase in each predictor (per 10 percentage points for percentage-based variables, per $100.00 for monetary measures, and per 100 events for conflict). Given the panel size of two countries (*G* = 2), *p*-values were calculated using a *t*-distribution with 1 degree of freedom. Interpretation focuses on effect sizes and Driscoll-Kraay 95% CIs.

TB, tuberculosis; HIV, human immunodeficiency virus; GDP, gross domestic product; CI: confidence interval; SE, standard error; DK, Driscoll-Kraay (standard errors).

#### Sensitivity analyses and robustness checks

The results from the linear mixed-effects models and OLS with HC3 standard errors were in agreement with those of the main analysis. Gross domestic product per capita continued to show a negative association with TB incidence, and TB-HIV co-infection also remained inversely associated with TB incidence in these models. The findings were robust across different estimation methods. Additional details can be found in Online Appendix 1 Table 3-A1.

#### Supplementary mortality model, 2000–2022

When examining TB mortality excluding deaths attributable to HIV, TB-HIV co-infection was found to be negatively associated with TB mortality (*b* = –2.138, Driscoll-Kraay 95% CI: –2.877 to –1.399). Gross domestic product per capita also demonstrated a negative association with TB mortality (*b* = –0.075, 95% CI: –0.102 to –0.048). The estimates for health expenditure were not precise, while conflict events showed a negative relationship with TB mortality (*b* = –0.041, 95% CI: –0.076 to –0.006). The full results are presented in Online Appendix 1 Table 4-A1.

### Synthesis of findings

Across all study periods and estimation approaches, higher GDP per capita consistently corresponded with lower TB incidence and mortality. These results align with the effect estimates shown in Table 3 and Table 4 and Online Appendix 1 Table 4-A1.

## Discussion

This retrospective ecological panel study compared the TB burden in Liberia and Sierra Leone over the period 2000–2022. Despite both countries facing post-conflict challenges, their trajectories differed. Liberia reported a prolonged increase in reported TB incidence followed by a subsequent plateau, whereas Sierra Leone experienced a steady decline. These trends suggest that variations in national recovery strategies, the effectiveness of service delivery, and the reliability of data systems may have contributed to the outcomes observed. No causal inferences are made, and the associations should be interpreted in light of the study’s design limitations.

In other conflict-affected and post-conflict settings beyond West Africa, national TB programmes have also had to rebuild surveillance and service delivery under severe constraints. Post-conflict Afghanistan, for example, expanded the directly observed treatment, short-course (DOTS) strategy and integrated TB treatment into primary care despite insecurity,^19^ achieving gains in case detection and treatment success as stability and funding improved. Experiences from Afghanistan and from protracted-conflict contexts such as Syria show that protecting routine TB services, investing in community-based case finding, and integrating TB and HIV care during recovery can help translate economic and health-system gains into sustained reductions in TB burden,^20,21^ lessons that are directly relevant for Liberia and Sierra Leone as they refine and scale up their TB-HIV programmes.

Across specifications, GDP per capita was consistently and inversely associated with TB incidence and with TB mortality, excluding HIV-attributable deaths. This pattern aligns with evidence that economic recovery and system capacity support better access to diagnosis and treatment and more reliable delivery systems.^2,3,12^ Tuberculosis-human immunodeficiency virus co-infection was negatively associated with TB incidence and mortality in several models. This likely reflects better case finding and reporting among people who access integrated TB-HIV services, rather than a protective effect. The small number of country clusters and variability in co-infection measurement warrant caution in interpreting these estimates.

### Tuberculosis-human immunodeficiency virus testing coverage and surveillance limitations

The analysis restricted to the 2016–2022 period did not identify a statistically significant link between TB-HIV testing coverage and TB incidence. The positive coefficient suggests that expanding testing may increase the number of detected cases in the short term, even if actual transmission rates remain unchanged. This finding aligns with recommendations that testing must be integrated with same-day treatment initiation, ongoing care and dependable commodity and information systems to achieve population-level improvements.^6^ In fragile contexts, incomplete reporting and delays in data collection can further diminish the strength of observed associations between testing and incidence.⁸

### Conflict exposure and tuberculosis outcomes

Conflict events did not demonstrate a significant association with TB incidence in most models. However, the supplementary mortality analysis indicated a possible inverse relationship. This result should be interpreted with caution, as it may reflect limitations in reporting, challenges in event classification or changes in health service utilisation during periods of instability. Previous research during and after the 2014–2016 Ebola outbreak documents the indirect effects of such shocks on service delivery, access to care and routine surveillance in Liberia and Sierra Leone.^7,8,9^

### Programmatic priorities for fragile contexts

Programmes should align testing scale-up with same-day linkage and treatment capacity, and track time to treatment and treatment completion as routine indicators. Data systems should keep pace with testing by using stable patient identifiers and regular reconciliation across TB and HIV registers. Laboratories and essential commodities should be protected during budget cycles and shocks, so detection gains are followed by reductions in transmission. These actions operationalise integrated TB-HIV care and reinforce core health-security functions.^2,5,6,7,10,11^

### Health-security perspective

A health-security approach focuses on the core functions that keep populations protected during times of shock. The negative relationship between GDP per capita and TB burden reflects gains in these functions. Examples include reliable laboratories, stable supply chains, protected health workers and connected surveillance. Timely feedback is also crucial.^5,6,7,10^ As these capacities improve, access to diagnosis and treatment becomes more dependable, and programme performance is easier to maintain.

Integrated TB-HIV services provide a practical framework for continuity. Shared workflows, common identifiers, harmonised registers, and coordinated commodity management help patients move from testing to treatment, even during disruptions. Whether facing outbreaks, staffing constraints or budget shortfalls, integration reduces drop-offs between case finding, linkage and treatment completion.^5,6,7,10^ The same infrastructure also supports rapid recovery once operations stabilise, strengthening system resilience. As programmes rebuild, improved case finding and reporting may increase the number of detected cases before incidence declines.

A health-security approach anticipates this pattern. It pairs scale up with investments in treatment capacity, laboratory throughput, information systems and routine feedback cycles. These steps align with core system-strengthening priorities. Together, they increase the likelihood that improved detection leads to sustained reductions in TB incidence and mortality.^5,6,7,10^

### Study limitations and future research

This analysis relies on publicly available surveillance and statistical model outputs that may carry measurement error, particularly when systems are disrupted. The number of country clusters is small, so inference is fragile, and emphasis is placed on effect sizes and CIs. Moreover, the study does not claim causal attribution. These limitations do not affect internal consistency, but they limit generalisability. Future research could apply this framework to additional fragile and conflict-affected contexts, examine variation in implementation of integrated TB-HIV services, and test whether improvements in linkage and data systems alter the relationship between expanded testing and reported incidence.

## Conclusion

This study indicates that a straightforward two-country panel approach can inform the evaluation of TB trends within fragile health systems. Liberia and Sierra Leone exhibited divergent trajectories, highlighting the interplay between economic conditions, co-infection dynamics and surveillance capacity. Higher GDP per capita was associated with a lower TB burden, whereas TB-HIV testing coverage between 2016 and 2022 was not linked to incidence. These findings underscore the need for metrics that monitor integration performance and linkage to treatment. The analytical framework employed here is replicable for assessing infectious disease control in post-conflict settings. Future research should apply this approach to additional contexts and prioritise equity, system resilience and sustained investment in public health infrastructure.
